# Patients’ understanding and perception of erectile dysfunction: Hong Kong versus Europe

**DOI:** 10.1007/s00345-025-05661-x

**Published:** 2025-05-04

**Authors:** Ruofan Shi, Stacia Tsun-Tsun Chun, Rong Na, Yan Zhang, Karl H. Pang

**Affiliations:** 1 Division of Urology, Department of Surgery, School of Clinical Medicine, LKS Faculty of Medicine, The University of Hong Kong, Hong Kong (HK), China; 2 Division of Urology, Department of Surgery, The University of Hong Kong-Shenzhen Hospital, Shenzhen, China; 3 Division of Urology, Department of Surgery, Queen Mary Hospital, Hong Kong (HK), China; 4 Department of Infertility and Sexual Medicine, The Third Affiliated Hospital, Sun Yat-Sen University, Guangzhou, China; 5 Division of Surgery and Interventional Science, University College London, Gower Street, London, WC1E 6BT UK; 6 Department of Andrology, University College London Hospitals NHS Foundation Trust, London, UK; 7 Department of Urology, Chelsea and Westminster Hospital NHS Foundation Trust, London, UK; 8 Consultant Urological Surgeon and Andrologist, Chelsea and Westminster Hospital, 369 Fulham Road, London, SW10 9NH UK

**Keywords:** Erectile dysfunction, Awareness, Hong Kong, Europe, European Association of Urology, The Urology Foundation

## Abstract

**Purpose:**

To estimate the prevalence of Erectile dysfunction (ED) in a Hong Kong (HK) population, evaluate the understanding and perception of ED and compare results with the European Association of Urology (EAU) and The Urology Foundation Trust (TRTed-TUF) data.

**Methods:**

A survey-based study was conducted between Dec 2023 and Mar 2024. The EAU and TRTed-TUF surveys were used as a template to design the current survey to ensure consistent reporting. Questions on the understanding of ED, healthcare seeking behaviour and awareness of treatment options were included. The survey was distributed via an online link to students and staff from The Hong Kong University (HKU) and to patients attending outpatient clinics at HKU Queen Mary Hospital.

**Results:**

616 responses were received from men aged 18–81 years. The prevalence of ED in our population was 51.8%. 53.2% (EAU 33.6%) were incorrect about what ED is, 54.0% (EAU 56.5%) knew that ED could be treated and 26.4% (TRTed-TUF 22%) were aware that ED could be a sign of heart disease. 73.6% (EAU, 26.3%) do not talk with anyone about ED. 88.3% (TRTed-TUF 77%) would be more likely to seek help if they knew ED was a sign of heart disease. Up to 32.6% (EAU 17.2%) have heard of alternative therapies to oral phosphodiesterase-5 inhibitors.

**Conclusions:**

ED awareness is low, and it is important to raise awareness possibly through charity campaigns, social media and education in primary care in order to detect underlying cardiovascular disease and reduce the impact of ED on psychosocial health.

**Supplementary Information:**

The online version contains supplementary material available at 10.1007/s00345-025-05661-x.

## Introduction

Erectile dysfunction (ED) is a common condition which may affect an individual’s physical, psychological and social health [1]. The Massachusetts Male Aging Study (MMAS) reported an overall ED prevalence of 52% in non-institutionalized men aged 40–70 years in the Boston area [2]. A study of ED from Hong Kong (HK) which included 1,506 men aged 26–70 years reported a prevalence of 36.7% [3]. ED may be a presenting complaint of an undiagnosed underlying cardiovascular disease (CVD), therefore early detection is important to prevent a significant cardiovascular event [4, 5]. Men who experience changes in their erections may not know what the underlying cause is, whether it is temporary or permanent, whether it is reversible or treatable and what treatment options are available.

Recent awareness surveys from TRTed and The Urology Foundation (TRT-ed-TUF) from the United Kingdom (UK) [6] and European Association of Urology (EAU) [7] demonstrated a low awareness of ED with regards to its definition, its association with heart disease and treatment options. In addition, the surveys revealed that around half of patients with ED would not see a doctor. The degree of awareness of ED in HK is unknown. Therefore, the aim of this survey-based study was to estimate the prevalence of ED in our study population and to explore patients’ understanding and perception of ED in HK, and to compare results with data from the TRTed-TUF and EAU.

Results would enable appropriate ED awareness campaigns to be initiated and encourage men to seek medical healthcare not only to diagnose and treat ED, but also to rule out any severe underlying causes and provide education on the natural history of ED and the various treatment options available.

## Methods

### Survey design

Following institutional review board approval from the University of Hong Kong (UW 23–474), a survey-based study was conducted. There are no validated questionnaires on ED awareness, therefore the current survey was designed using the EAU and TRTed-TUF surveys as a guide to ensure consistent reporting. The survey was designed using the online platform Qualtrics (https://www.qualtrics.com/en-gb/) and included questions on the understanding of ED, healthcare seeking behaviour and awareness of different types of treatments available. The 5-item International Index for Erectile Function (IIEF-5) questions [8] were also incorporated into the survey to assess the degree of ED within our study population. The survey introduction detailed the nature of the study and participants had the option of consenting to participate and proceeding with the survey, or not consenting and exiting the survey. The survey distributed in this study is shown in Supplementary file 1.

### EAU and TRTed-TUF surveys

The EAU survey was conducted in July 2020 and included 3,032 participants aged 20–70 years from Spain, France, Germany and the UK [7]. TRTed is a men’s health platform sharing trusted resources on men’s health issues. In collaboration with TUF, a survey of 2,000 men between 30th May and 6th June 2023 was conducted [6]. The age range of the participants in the TRTed-TUF survey was not stated. Both surveys focused on awareness, understanding and perception of ED.

### Survey distribution

A link to the survey was distributed electronically via the Hong Kong University (HKU) staff and students email portal. A barcode of the survey was also generated and distributed randomly to patients who were waiting in the waiting area for their outpatient clinic appointment from different specialties (medical or surgical) at HKU Queen Mary Hospital. This approach aimed to ensure that the sample was not biased towards individuals with andrological issues, allowing us to more accurately estimate the prevalence of ED in a broader population.

The online survey was opened between 01 December 2023 and 01 March 2024. All participants age *≥* 18 years who provided informed consent via the survey introduction were included.

### Data analysis and reporting

Results were analysed using Qualtrics and Microsoft Excel version 16. Data was reported as whole numbers and percentages. The checklist for reporting results of internet E-surveys (CHERRIES) [9] was used as a template for data reporting.

## Results

Overall, 616 responses from participants aged 18 and 81 years were received. A total of 489 (79.4%) responses were from HKU students and staff who completed the online survey sent to them by email, and 127 (20.6%) responses were from patients attending outpatient clinics who completed the survey by scanning a barcode on their personal phone. The prevalence of ED in the current cohort of patients was 51.8%. Overall, 34.6%, 8.2%, 7.3% and 1.8% had mild (IIEF-5 score 17–21), mild-moderate (12–16), moderate (8–11) and severe (5–7) ED respectively.

### Understanding of ED

**Fig. 1:**
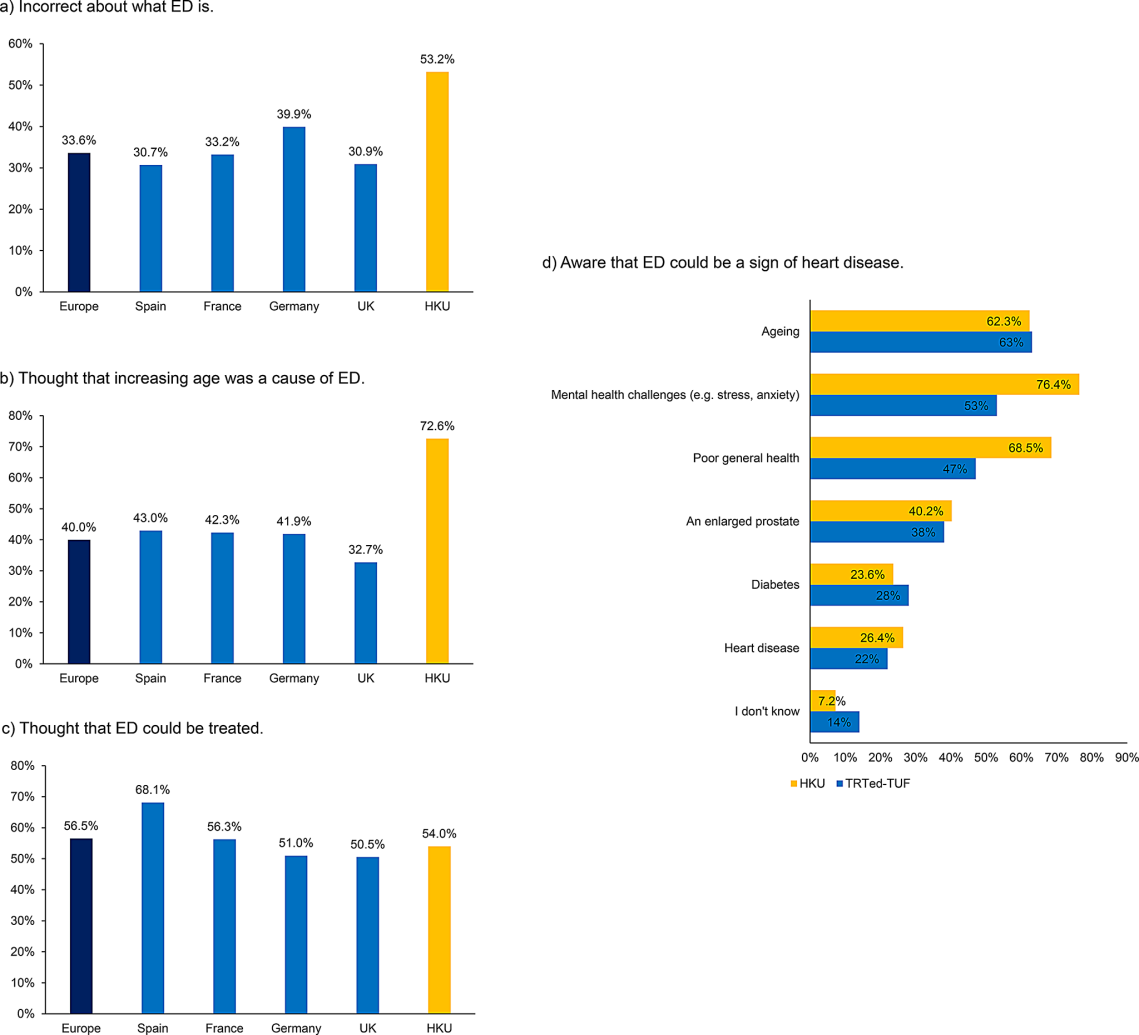
Understanding of erectile dysfunction. ED, erectile dysfunction; UK, United Kingdom; HKU, Hong Kong University

A total of 53.2% of participants (EAU, 33.6%) were incorrect about what ED is (Fig. 1a **and** Table 1a). 72.6% (EAU, 40.0%) thought that increasing age is the main cause of ED (Fig. 1b **and** Table 1b) and 54.0% (EAU, 56.5%) thought that ED could be treated (Fig. 1c **and** Table 1c). 26.4% (TRTed-TUF, 22%) were aware that ED could be a sign of heart disease (Fig. 1d).

**Table 1:** Understanding of erectile dysfunction

	EAU survey					Current
	Europe (all)	Spain	France	Germany	UK	HKU
**a) What do you think ED is?**						
Not able to get or keep an erection	64.8%	77.8%	66.5%	48.9%	66.0%	96.7%
Not able to get an orgasm/ejaculation	19.1%	20.4%	18.6%	20.9%	16.6%	40.9%
I don’t know	17.3%	10.1%	17.5%	25.8%	16.0%	1.1%
Incontinence	5.1%	3.8%	4.7%	6.8%	5.1%	4.3%
A constant need to urinate	4.9%	3.7%	5.4%	6.8%	4.0%	4.7%
Not able to urinate	4.0%	2.2%	4.1%	4.9%	4.9%	2.9%
Other, please specify	0.5%	0.7%	0.4%	0.5%	0.3%	0.4%
**b) What**,** if anything in particular**,** do you think can cause ED?**						
Psychological conditions like stress, anxiety, depression, relationship problems	47.7%	50.8%	51.8%	50.3%	37.6%	85.9%
Increased age	40.0%	43.0%	42.3%	41.9%	32.7%	72.6%
Consuming too much alcohol	38.1%	36.2%	38.3%	42.3%	35.6%	58.8%
Drug use	32.0%	30.5%	34.7%	34.8%	28.1%	50.5%
Medical conditions like cardiovascular disease, diabetes, high blood pressure, high cholesterol, MS or Parkinson’s disease	31.6%	29.8%	34.4%	32.3%	29.8%	53.1%
Certain prescription medications	30.2%	24.3%	34.0%	37.7%	25.0%	50.5%
Low testosterone levels or other hormone imbalances	29.8%	33.4%	27.1%	32.8%	25.8%	67.1%
Obesity	24.5%	20.5%	24.2%	28.1%	25.1%	56.3%
Using tobacco products	24.1%	19.6%	30.4%	27.4%	19.1%	61.7%
Sleep disorders	16.3%	12.4%	18.8%	19.7%	14.1%	40.4%
Not sure	15.1%	11.7%	9.7%	13.4%	25.5%	2.2%
Genetics	13.4%	9.5%	13.2%	16.6%	14.4%	40.4%
Kidney disease	8.9%	6.9%	9.9%	11.3%	7.4%	33.9%
Nothing in particular can cause ED	4.4%	4.0%	5.5%	2.6%	5.5%	5.1%
Other, please specify	0.9%	0.5%	0.9%	0.9%	1.1%	0.4%
**c) Do you think that you just have to accept living with ED or that it can be treated?**						
It can be treated	56.5%	68.1%	56.3%	51.0%	50.5%	54.0%
You just have to accept living with ED	9.1%	6.8%	10.0%	9.3%	10.4%	3.6%
Neither	6.7%	4.6%	8.2%	6.4%	7.8%	
Depends on the cause	15.0%	8.6%	19.0%	18.9%	13.6%	34.8%

EAU, European Association of Urology; ED, erectile dysfunction; MS, multiple sclerosis; UK, United Kingdom; HKU, Hong Kong University

### Healthcare seeking behaviour

**Fig. 2:**
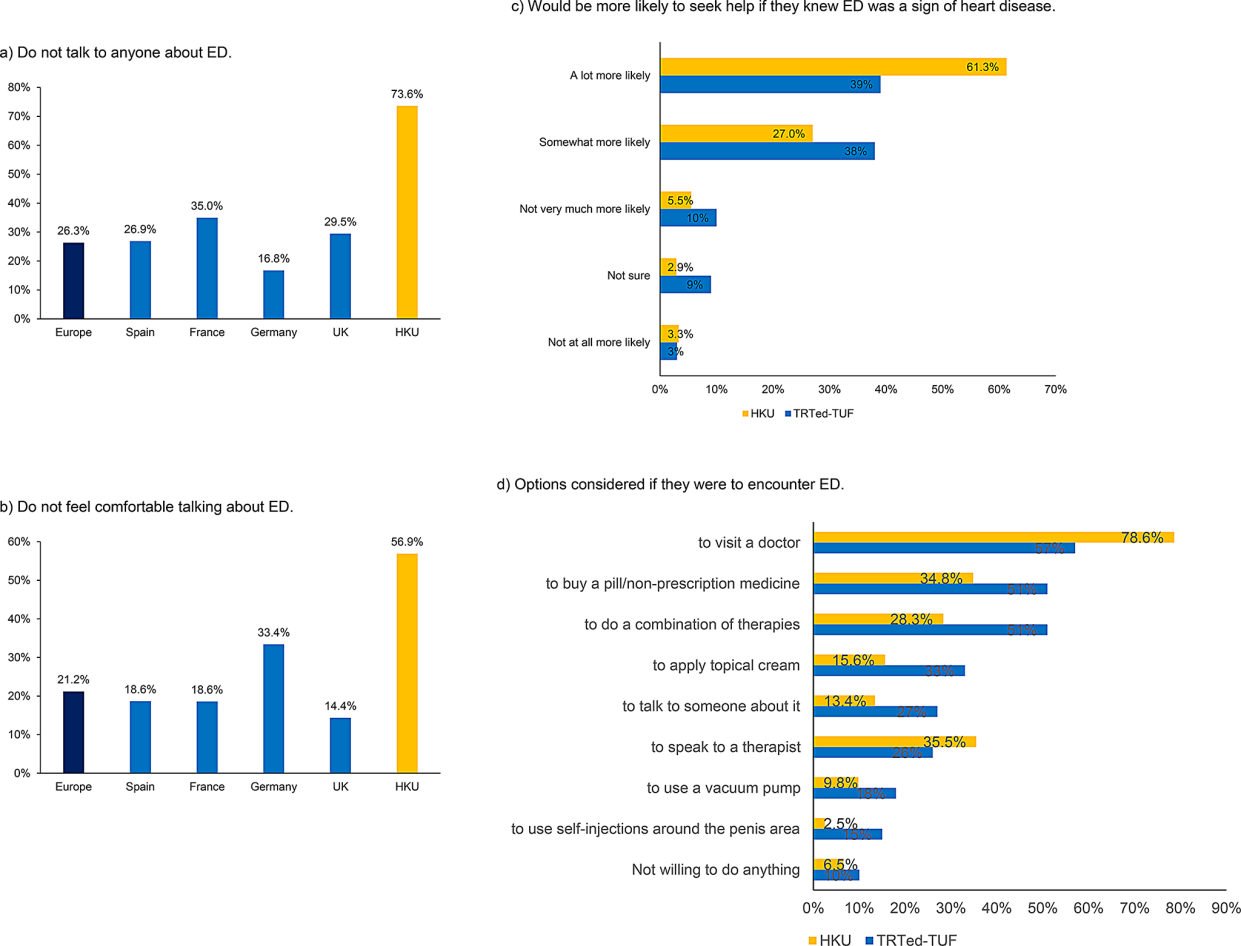
Understanding of erectile dysfunction. ED, erectile dysfunction; UK, United Kingdom; HKU, Hong Kong University

Amongst individuals with ED, 73.6% (EAU, 26.3%) do not talk with anyone about their experience with ED and 56.9% (EAU, 21.2%) do not feel comfortable talking about ED (Table 2**and** Fig. 2a and b). 88.3% (TRTed-TUF, 77%) would be somewhat more or a lot more likely to seek help if they knew ED was a sign of heart disease (Fig. 2c). If participants were to encounter ED, 78.6% (TRTed-TUF, 57%) would visit a doctor, 34.8% (TRTed-TUF, 51%) would buy a pill and 2.5% (TRTed-TUF, 15%) would use a penile injection (Fig. 2d).

**Table 2:** Healthcare seeking behaviour. If you have ever experienced erectile dysfunction, did you talk with someone about it?

	EAU survey					Current
	Europe (all)	Spain	France	Germany	UK	HKU
Yes, to each other	30.7%	33.6%	30.1%	37.9%	19.4%	57.1%
No	26.3%	26.9%	35.0%	16.8%	29.5%	73.6%
Yes, with my GP	25.4%	25.4%	28.5%	19.9%	29.5%	21.4%
Yes, a urologist or sexologist	16.8%	23.9%	14.6%	20.5%	7.0%	21.4%
Yes, a sexual therapist or psychologist	10.8%	11.9%	8.1%	11.8%	10.9%	7.1%
Yes, with (a) friend(s)	9.3%	10.4%	6.5%	14.3%	4.7%	50.0%
Yes, with family	7.3%	8.2%	4.1%	11.2%	4.7%	7.1%
Yes, with someone else not listed	7.1%	6.0%	6.5%	8.1%	7.8%	7.1%
Prefer not to say	1.3%	0.7%	0.8%	0.0%	3.9%	7.1%

UK, United Kingdom; HKU, Hong Kong University

### Awareness of ED treatments

A total of 83.7% (EAU, 54%) have heard of oral phosphodiesterase-5 inhibitors (PDE5i) and up to 32.6% (EAU, 17.2%) have heard of other treatment options such as vacuum devices, penile injections, topical therapies, shockwave therapy and penile implants (Table 3).

**Table 3:** Understanding of erectile dysfunction treatment options. Which, if any, of the following treatments for ED have you ever heard of?

	EAU survey					Current
	Europe	Spain	France	Germany	UK	HKU
Medication (i.e. Viagra, Cialis, Levitra, Stendra)	54.0%	61.1%	53.6%	52.8%	48.4%	83.7%
None of the listed	26.4%	17.9%	29.1%	27.3%	31.4%	5.1%
Sexual education and relationship therapy	24.0%	38.4%	19.6%	19.7%	18.2%	49.3%
Vacuum erection device	17.2%	12.8%	19.6%	20.0%	16.6%	32.6%
Penile injections	14.3%	14.9%	14.0%	13.6%	14.9%	22.8%
Penile implants	11.3%	8.1%	10.7%	13.5%	13.0%	29.0%
Shockwave therapy for ED	7.3%	10.7%	3.7%	8.5%	6.3%	11.2%
Topical therapies	6.8%	9.9%	2.5%	6.9%	7.7%	15.2%

ED, erectile dysfunction; UK, United King_dom; HKU, Hong Kong University

## Discussion

In this current study, the EAU and TRTed-TUF awareness of ED questionnaires were used as a reference to formulate the current survey. The aim was to estimate the prevalence of ED in our study population and to understand the patient’s awareness and perception of ED in HK compared to Europe.

The prevalence of ED in our study population was 51.8%. The MMAS study reported an ED prevalence of 52% in men aged 40–70 years [2]. A survey-based study of ED in 8 countries (Italy, France, China, Spain, Germany, USA, UK, Brazil), which included 97,159 patients between the age of 40 and 70 years demonstrated a similar prevalence of 42.1–52.2% [10]. A study which included 1,506 men from HK reported a slightly lower prevalence of 36.7% [3]. The prevalence of ED in our study cohort is within the range reported in the literature.

Our results indicated that in general, the awareness and perception trend was similar in both the cohorts in HK and Europe. Around half of the participants in HK (53.2%) and a third in Europe (EAU, 33.6%) were incorrect about what ED is, and around 5% of participants in HK and Europe thought ED was some form of urinary symptom. Lower urinary tract symptoms (LUTS) may occur with ED, and this may cause confusion amongst patients with regards to the medical definition of ED or LUTS. Data from multinational surveys and USA benign prostate hyperplasia (BPH) registries, suggested that around 50–70% of men who have LUTS also have ED [11, 12]. Therefore, it is important to educate patients and the public what ED actually is and that this condition is not urinary in nature and the treatment of ED and LUTS are completely different.

Just over half of participants in HK (54%) and Europe (EAU, 56.5%) were aware that ED could be treated. Importantly, a small proportion of around quarter of participants in HK (26.4%) and UK (TRTed-TUF, 22%) were aware that ED could be a sign of heart disease. It is known that ED could be an early manifestation of CVD [4, 5] and a meta-analysis which included 154,794 individuals demonstrated that the CVD, coronary heart disease and stroke risk of ED patients was significantly increased by 43%, 59% and 34% respectively compared with men without ED [4]. In addition, the all-cause mortality increased by 33% [4]. Therefore, it is important to deliver the message that ED may be associated with CVD, it is important to investigate ED, and that ED could be treated.

When enquired about healthcare seeking behaviour, it appeared that the percentage who do not talk with anyone about ED was much higher in HK (73.6%) than in Europe (EAU, 26.3%). The reason for this difference is not entirely clear, however this may be due to the fact that more participants in HK (56.9%) felt less comfortable with talking about this issue compared to participants from Europe (EAU, 21.2%). Possible stigma or embarrassment of having ED may lead to denial of the issue, not considering it as a medical condition and preventing one from discussing ED with anyone.

Majority of participants would visit a doctor or buy a pill if they were to encounter ED, and over three quarters of participants in HK (88.3%) and UK (TRTed-TUF, 77%) cohorts would be more likely to seek help for their ED if they knew that it was a sign of heart disease. Therefore, patients and the public need to be encouraged to come forward with their sexual health problems and be made aware that treatments are available and that screening of underlying causes and associated disease such as CVD would be undertaken.

Majority of the participants have heard about PDE5i, however under a third of participants in HK (32.6%) and under a quarter in Europe (EAU, 17.2%) have heard of other treatments such as vacuum devices, topical agents, intracavernosal injections, shockwave therapy and penile implants. The low awareness of treatment options may explain why only around a third of participants would consider other alternative treatment options to PDE5i if they were to encounter ED.

It is important to raise public awareness of ED as a possible early manifestation of CVD. The public and affected individuals need to understand that men of any age could be affected and that it could be treated, and many options are available. Apart from oral and the current intracavernosal pharmacotherapies, there are increasing evidence on regenerative and novel non-surgical options. These include intracavernosal platelet-rich plasma [13] and botulinum neurotoxin [14] injections and shockwave therapy [15]. Awareness needs to be raised to exclude severe underlying diseases and reduce psychosocial consequences on affected individuals and their partners. Possible strategies to improve awareness include charity campaigns, the use of social media and asking screening questions and providing appropriate education where possible in primary care.

### Limitations

This study did not represent the whole of HK but provided an estimate of the prevalence of ED and degree of awareness and understanding in a cohort of HK individuals from HKU and Queen Mary Hospital outpatients. Most of our survey links were disseminated via bulk email, so we could not estimate the exact number of recipients who received and engaged with the invitation and how many patients were in the outpatient waiting area, therefore, the response rate was not calculated.

In conclusion, the 51.8% prevalence of ED in the current study population is within the range reported in the literature. Consistent with surveys from Europe, the awareness of ED is low, and this could possibly be improved by hosting charity campaigns and improving education via social media and primary care.

## Electronic supplementary material

Below is the link to the electronic supplementary material.

Supplementary Material 1

## Data Availability

No datasets were generated or analysed during the current study.

## Declarations

### Registry and the registration no. of the study/trial

N/A.

### Animal studies

N/A.
