# Supplementary material for: Patients’ understanding and perception of erectile dysfunction: Hong Kong versus Europe
Source: World J Urol. 2025 May 4;43(1):263. doi: 10.1007/s00345-025-05661-x (PMC12050226; doi:10.1007/s00345-025-05661-x)
Supplement: Supplementary file 1 — Supplementary Material 1 [file 345_2025_5661_MOESM1_ESM.docx]

**Contemporary awareness, prevalence and severity of Erection Dysfunction in Hong Kong**

*Patient Informed Sheet*
Your participation the study is entirely voluntary. You are free to withdraw at any time without giving any reason. This will not affect the standard of care you receive.

*What is the purpose of this study?*

The aim of this study is to establish the contemporary awareness, prevalence and severity of Erectile Dysfunction (ED) in a Chinese population within Hong Kong. Results may help improve awareness of ED and its treatment, encourage patients with ED to seek medical advice and management.

*Why am I being chosen and what will happen to me if I take part?*
Any Chinese male aged >18 years with ability to give consent is eligible for this study. You will be invited to complete a questionnaire that takes round 10-20 minutes.

.
*What will be the risk to me if I take part in this study?*

The study is based on questionnaire only and will not impose any risks.

*What will happen to the results of the research study?*

The results of the study will be used for research purposes only. Data from individual patients will be treated with the strictest confidentiality. The identity of the patients will not be identifiable in any reports or publications.

*By consenting to participate in this study, you expressly authorize:*

the principal investigator and his research team and the ethics committee (Institutional Review Board of the University of Hong Kong/ Hospital Authority Hong Kong West Cluster) are responsible for overseeing this study to get access to, to use, and to retain your personal data for the purposes and in the manner described in this informed consent process; and the relevant government agencies (e.g. the Hong Kong Department of Health) to get access to your personal data for the purposes of checking and verifying the integrity of study data and assessing compliance with the study protocol and relevant requirements.

*Study:* Contemporary awareness, prevalence and severity of Erectile Dysfunction and in the Chinese population within Hong Kong.

Centre: Department of Surgery, Li Ka Shing Faculty of Medicine University of Hong Kong Principal Investigator: Dr. Karl Ho Pang

- I confirm that I have read and understood the information sheet for the above study and have my questions adequately answered. I understand that my participation is voluntary and that I am free to withdraw at any time, without giving any reason, without my medical care or legal rights being affected. **I agree to take part in the above study.** (1)
- I do not agree to take part in the above study. (2)

How old are you?

________________________________________________________________

What do you think erectile dysfunction (ED) is?

- Not able to get an erection (1)
- Not able to ejaculate/orgasm (2)
- I don’t know (3)
- Urinary incontinence (4)
- A constant need to urinate (5)
- Not able to urinate (6)
- Other_________________ (7)

What, if anything in particular, do you think can cause ED?

- Psychological conditions like stress, anxiety, depression, relationships problems (1)
- Increased age (2)
- Consuming too much alcohol (3)
- Drug use (4)
- Medical conditions such as cardiovascular disease, diabetes, high blood pressure, high cholesterol, MS or Parkinson’s disease (5)
- Certain prescription medications (6)
- Low testosterone or other hormone imbalance (7)
- Obesity (8)
- Tobacco products (9)
- Sleep disorders (10)
- Not sure (11)
- Genetics (12)
- Kidney disease (13)
- Nothing in particular can cause ED (14)
- Pelvic surgery or radiation (15)
- Other, please specify________

What do you think the persistent difficulties getting and/or maintaining an erection could be a sign of?

- Ageing (1)
- Mental health challenges (e.g. stress, anxiety) (2)
- Poor general health (3)
- An enlarged prostate (4)
- Diabetes (5)
- Heart disease (6)
- I don't know (7)

Do you think that you just have to accept living with ED or that it can be treated?

- It can be treated (1)
- You just have to accept living with ED (2)
- Neither (3)
- Not sure (4)
- Depends on the cause (5)

Which, if any, of the following treatments for ED have you ever heard of?

- Medications (e.g. viagra, cialis) (1)
- Life style intervention (2)
- Sexual education and relationship therapy (3)
- Vacuum erection device (4)
- Penile injection (e.g. caverject) (5)
- Penile implants (surgical implantation of prosthesis) (6)
- Shock wave (7)
- Topical or urethral medications (e.g. alprostadil) (8)
- None of the above (9)
- Others not listed, please specify (10)_______

Which, if any, of the following options would you consider fixing any erectile dysfunction problems you may encounter?

- Willing to visit a doctor (1)
- Willing you buy a pill/non-prescription (2)
- Willing to do a combination of therapies (3)
- Willing to apply topical cream (4)
- Willing to talk to someone about it (5)
- Willing to speak to a therapist (6)
- Willing to use a vacuum pump (7)
- Willing to use self-injections (8)
- Willing to undergo penile prosthesis surgery (9)
- Not willing to do anything (10)

If persistent failure to obtain/ maintain an erection was an officially recognised sign of heart disease, how much more likely would you be to visit your doctor to raise concerns about any erectile function issues?

- A lot more likely (1)
- Somewhat more likely (2)
- Not very much more likely (3)
- Not sure (4)
- Not at all more likely (5)

How do you rate your confidence that you could get and keep an erection?

- Very low (1)
- Low (2)
- Moderate (3)
- High (4)
- Very high (5)

When you had erections, how often were the erections hard enough for penetration?

- No sexual activity (1)
- Almost never (2)
- Few times (much less than half) (3)
- Moderate Sometimes (about half the time) (4)
- Most times (much more than half the time) (5)
- Almost always/ always (6)

During sexual intercourse, how often were you able to maintain your erection after you had penetrated (entered) your partner?

- Did not attempt intercourse (1)
- Almost never (2)
- A few times (much less than half) (3)
- Sometimes (about half the time) (4)
- Most times (much more than half the time) (5)
- Almost always/ always (6)

How difficult was it to maintain your erection to completion of intercourse during sexual intercourse?

- Did not attempt intercourse (1)
- Extremely difficult (2)
- Very difficult (3)
- Difficult (4)
- Slightly difficult (5)
- Not difficult (6)

How often was your erection satisfactory when you attempted sexual intercourse?

- Did not attempt intercourse (1)
- Almost never (2)
- A few times (3)
- Sometimes (4)
- Most times (5)
- Almost always/ always (6)

If you did not seek medical advice, what were the reasons? Check all that apply

- Think That it was normal (1)
- Did not know there is treatment for this condition (2)
- Embarrassment (3)
- Did not think it was a serious medical condition (4)
- Did not have time (5)
- Family or general practice doctor did not want to address the issue or refer to a specialist (6)

Did you discuss your erection issues with others?

- Yes (1)
- Friends (2)
- Partner (3)
- Family members (4)
- Family doctor (5)
- Urologist (6)
- Sexual therapist or psychologist (7)
- No one (8)
- Prefer not to say (9)
- Others, please specify (10)____________________
